# Engaging Social Stakeholders in National Asbestos Research for Public Health: An Italian Experience

**DOI:** 10.5334/aogh.4717

**Published:** 2025-06-05

**Authors:** Daniela Marsili, Alessandra Binazzi, Alessandro Marinaccio, Carolina Mensi, Lucia Fazzo, SEPRA Project Working Group for the Study on Communication and Social Stakeholders’ Engagement

**Affiliations:** 1 Environmental and Social Epidemiology Unit, Department of Environment and Health, Istituto Superiore di Sanità, Rome, Italy; 2 WHO Collaborating Centre for Environmental Health in Contaminated Sites, Rome, Italy; 3 Department of Occupational and Environmental Medicine, Epidemiology, Hygiene, National Institute for Insurance against Accidents at Work (INAIL), Rome, Italy; 4 Occupational Health Unit, Fondazione IRCCS Ca’ Granda Ospedale Maggiore Policlinico, Milan, Italy

**Keywords:** environmental public health communication, stakeholders’ engagement, asbestos, asbestos‑related diseases, mesothelioma, prevention, health equity, participatory research

## Abstract

*Background:* Asbestos and its impacts on the population worldwide are threats to the environment and public health, striking both countries that have banned asbestos, such as Italy in 1992, and those that continue its use. In Italy, asbestos‑affected communities have experienced diverse progress in their involvement in prevention and asbestos risk management.

*Objectives:* Exploring social stakeholders’ engagement in research projects on asbestos health impacts and discussing the benefits of the ongoing SEPRA project (Epidemiological Surveillance, Prevention and Research on Asbestos).

*Methods:* A structured communication plan was implemented, including the selection of social stakeholders and their engagement during the project’s lifetime; selection of research topics to characterize the participative initiatives and communication modality; and collection and analysis of inputs for enabling impacts.

*Findings:* Sharing major issues of concern highlighted by social stakeholders, such as the recognition of all asbestos‑related diseases besides mesothelioma and non‑occupational asbestos exposures. The need to implement health surveillance plans in all the regions of the country, including a national plan addressing the psychological support to patients and their families, and to extend social welfare to people affected by occupational and non‑occupational asbestos‑related diseases has been highlighted.

*Conclusions*: Social stakeholders’ engagement in research activities through structured interactions and trusted relationships allowed us to share information, needs and recommendations for implementing effective actions in public health. Researchers committed to asbestos research at a national scale should closely collaborate with stakeholders through structured communication, considering their diversified fields of experience, critical thinking and inputs. Giving recognition to social stakeholders of their role and expertise and providing them appropriate tools to interact with the relevant authorities and the asbestos‑affected communities are key for effectively advancing in inclusive processes and health equity.

## Introduction

Asbestos and its impacts on the population worldwide are key issues for global environmental and public health, striking both countries that have banned asbestos and those that continue its use [1–7]. In 2012, the International Agency for Research on Cancer (IARC) confirmed the evaluation of carcinogenicity of asbestos, stating that all forms of asbestos cause mesothelioma and cancers of the lung, larynx, and ovary; a positive association is reported for pharynx, stomach, and colorectum cancers [8]. Furthermore, asbestos causes non‑oncological diseases, i.e. asbestosis and pleural plaques. In 2017, IARC defined that exposure to fluoro‑edenite, an amphibole fibre, also causes mesothelioma with sufficient evidence [9].

Among the actions required to eradicate asbestos‑related diseases (ARDs), the promotion of environmental awareness via education, the implementation of joint actions among societal actors, governmental and non‑governmental organizations are particularly appropriate. Furthermore, interdisciplinary research identifying the impact of diseases caused by asbestos and the mapping of asbestos exposure sources are needed [10].

Environmental, health, and social impacts of asbestos are still present today in Italy, more than 30 years since the asbestos ban entered into force in 1992 [11, 12]. This is due to the long latency of ARDs and to the residual exposure to asbestos still in place, also caused by the delay in remediation of asbestos‑contaminated sites, as reported in the countries that banned asbestos in the past [13, 14].

The contrast and eradication of asbestos exposure sources and ARDs are recommended by International Labour Office and World Health Organization and are among the priorities for achieving the goals of 2030 United Nations’ Agenda for Sustainable Development [15, 2], and the implementation of epidemiological surveillance plans of ARDs is recommended in the National Asbestos Plans [15, 6].

In Italy, a national epidemiological plan for mesothelioma mortality surveillance has been ongoing since the early 1990s, and a National Mesothelioma Registry (ReNaM) has collected all incidence cases since 2002, through the Regional Operating Centres (COR‑ReNaM) [16]. The periodic updates of the national epidemiological surveillance of mortality [12, 17–19] and ReNaM [16] provide data and evidence‑based information on asbestos risks and health impacts. Communication of results with institutional stakeholders (i.e. Ministry of Health, Ministry of Environment, national and local policymakers) and local social stakeholders (i.e. environmental non‑governmental organizations (NGOs), associations of former asbestos exposed and their relatives, and trade unions) has characterized the epidemiological surveillance systems since the beginning to foster knowledge and awareness of the health impact of asbestos in population.

Furthermore, several epidemiological studies on single asbestos‑contaminated sites and workers’ cohorts included the communication of results and the engagement of local institutional and social actors in their activity plans. Their engagement has proven to be crucial for adopting prevention actions including the reduction/elimination of asbestos contamination sources affecting communities [20–22] as experienced in Casale Monferrato, a municipality in Northern Italy where one of the largest European asbestos‑cement plants operated until 1986 [23–25]. This stakeholders’ engagement is not common to other asbestos‑contaminated communities in Italy that experienced only minor progress in community involvement in prevention, remediation, and asbestos risk management [23], such as the asbestos‑affected communities of Broni in Lombardy [26] and San Cataldo in Sicily [12, 19].

The ongoing SEPRA project—Epidemiological Surveillance, Prevention and Research on Asbestos—is strongly rooted in public health connected to asbestos health impact. It is promoted and funded by the National Institute for Insurance against Accidents at Work (INAIL) and coordinated by the Fondazione IRCCS Ca’ Granda Ospedale Maggiore Policlinico, Milano, with the participation of the National Health Institute (Istituto Superiore di Sanità) and academic Institutions (Universities of Padua and of Turin). It integrates multidisciplinary knowledge and expertise at the national level with the aim to provide updated research and epidemiological surveillance systems of mortality and incidence cases for mesothelioma and the other ARDs, guidelines for registering mesothelioma cases in the ReNaM and assessing their exposure history, tools for healthcare assistance, psychological approaches to support patients and their caregivers, and recommendations for social policies concerning ARDs [27].

In the public health and equity perspective, the SEPRA project adopts a participative communication approach. It relies on bi‑ and multidirectional communication with stakeholders to strengthen relationships and exchanges among researchers and institutional and social stakeholders committed to the asbestos issues at the national and local levels, through their engagement in ongoing research activities. The project emphasizes an approach to communication with stakeholders that does not see communication as merely “passing of information” and it does not focus only on the content of conveyed messages about asbestos, health and risks. Conversely, social stakeholders’ engagement at the national scale throughout the lifetime of a multidisciplinary asbestos research project is essential because they can bring experiences in the research activities at both the national and local levels concerning practices to address the social and health‑related impacts on the asbestos‑affected population [28–31].

This paper aims to explore the involvement of stakeholders in national research projects on the health impacts of asbestos. In particular, we focus on social stakeholders’ engagement, and we discuss their inputs provided during the execution of the research activities planned in the SEPRA project operating on a national scale. We highlight the benefits for the project from the interactions with the engaged social stakeholders as well as the recognition of their role in representing the specific needs of impacted communities, strengthening the public health perspective of the envisioned research. We finally discuss the importance of this experience contributing to the implementation of participative public health recommendations to decision‑makers aimed at improving asbestos prevention and social policies in the equity perspective.

## Methods

### Conceptual framework

The communication approach used in this study relies on the conceptual framework designed by the Istituto Superiore di Sanità to implement environmental public health communication in contaminated sites. The communication framework has been proposed in international and national contexts for implementing structured communication plans as a key element of epidemiological studies and surveillance programmes of the population resident in contaminated sites (including those with asbestos exposure) [32, 33]. The communication plan is designed to consider the social characteristics of the impacted communities, as well as the type of contamination source and the related environmental and health impacts [33]. It foresees the “identification and engagement of relevant stakeholders” as one of the fundamental methodological steps to be implemented throughout the project period to enhance the application of the study findings for public health [32, 33].

In the SEPRA project operating at the national scale, we focus on the engagement of social stakeholders committed to asbestos social and health‑related issues in Italy. Differently from previous initiatives, we include both local and national social stakeholders as the key categories relevant for the participative communication activities.

**Figure 1:**
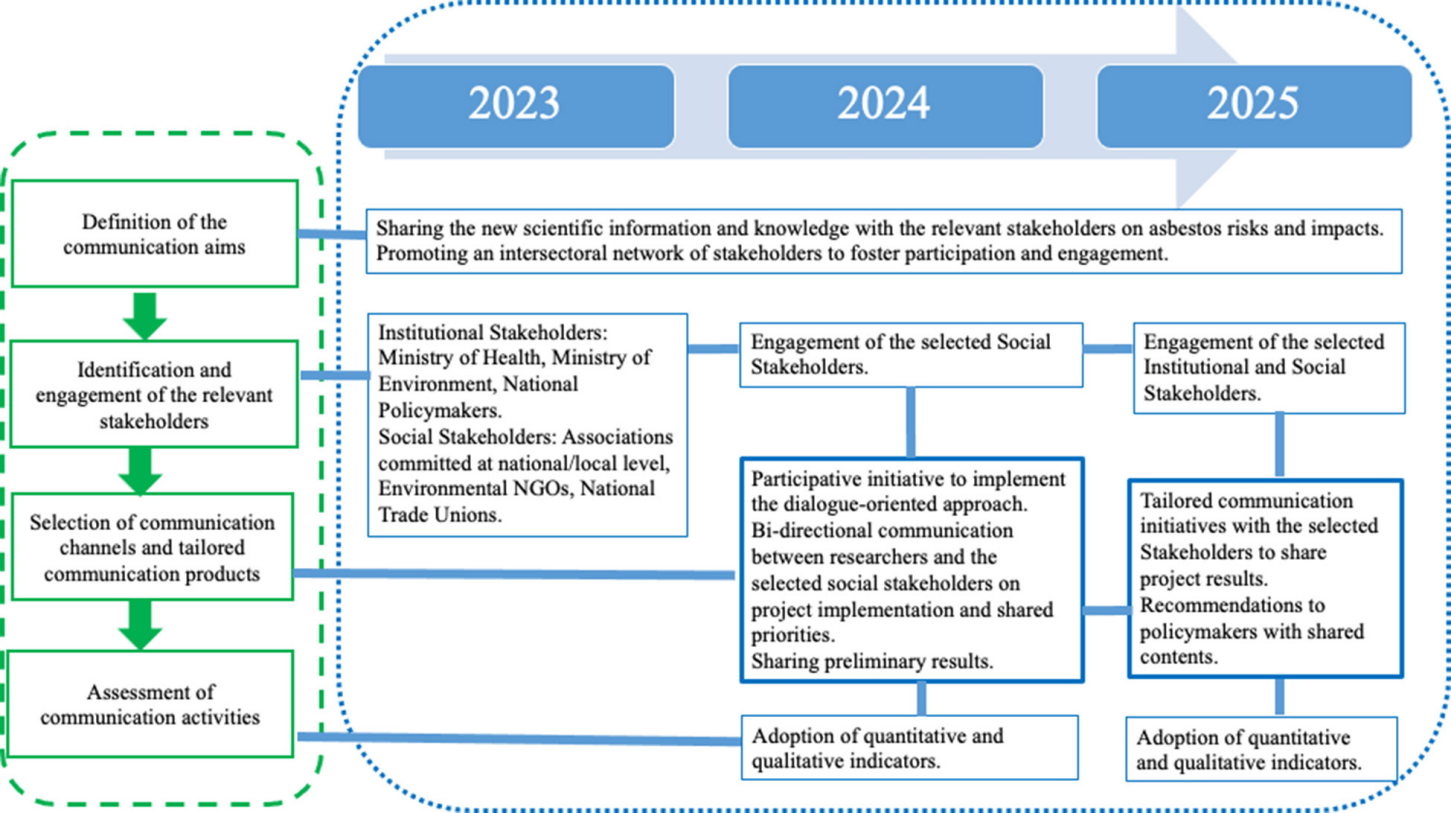
Implementation of the communication approach in the SEPRA project. The four methodological steps of the communication plan are depicted on the left panel (green dashed box). Its implementation in the SEPRA project is illustrated in the right panel (blue dotted box). The key actions are described in the blue rectangular boxes. The key actions involving bidirectional communication are described in the two blue boxes highlighted in bold. The project timeline is shown on the top of the figure. Blue lines depict the workflow of the implemented communication activities, undertaken coherently with the methodology and the timeline.

Figure 1 illustrates the architecture of the structured communication plan adopted in the SEPRA project. It shows the roadmap and the key actions envisioned to implement the plan. The step “identifying and engaging relevant stakeholders” considers the national and local social stakeholders as the key categories to be engaged during the project lifetime. The key actions relying on bidirectional communication between researchers and social/institutional stakeholders are envisioned in two specific stages, mid‑term and at the end of the project (blue bold boxes). Following the adopted methodology, impact assessment of communication activities is envisioned during the project lifetime.

### Selection of social stakeholders

We selected several categories of social stakeholders at the beginning of the SEPRA project to be engaged during the project lifetime, because of their long‑lasting lived experience in national and local contexts affected by asbestos exposure and health‑related impacts, as well as for their commitment to supporting affected people in addressing ARDs. Three categories of social stakeholders were selected:

- Associations of former asbestos‑exposed workers and Associations of patients and their families.
- Environmental NGOs.
- National trade unions committed to supporting asbestos‑exposed workers, patients, and their families for the social assistance and legal recognition of asbestos exposure.

### Engagement of the selected social stakeholders

The original methodological contribution implemented in the SEPRA project was the engagement of social stakeholders during the project’s lifetime with the aim to enable the participative discussion of the project activities and preliminary results, fostering the exchange of experiences useful to strengthen and better focus the subsequent project’s activities. This action represented a novel implementation of an asbestos research project in Italy operating at the national scale.

Participated communication and exchanges with social stakeholders on the research topics, which are considered particularly relevant for them, have been planned because of the implications in asbestos prevention and public health. The proactive involvement of social stakeholders during the project’s mid‑term phase was considered crucial for this study and represents a distinctive feature of the SEPRA project. In this regard, a focused workshop was held at the project’s midpoint to allow the project researchers and the involved social stakeholders to exchange and share knowledge, experiences, and collect mutual feedback on the ongoing project activities.

### Selection of research topics for the engagement

The social stakeholders need to be engaged in those specific topics involving their role and responsibilities, as well as their activities. The involvement should foresee the collection of feedback on the selected topics for fostering interactions, collaborations, and participative roles in project activities.

Discussion and feedback were focused on the primary objectives of the project and its initial outcomes [27].

- Updating the epidemiological surveillance of mortality from mesothelioma in Italy at the municipal level in 2010–2020 period: the analysis of the temporal trend since 2003 showed a slight decrease, particularly in individuals aged 50 years or younger. In 334 municipalities among men and 156 among females, a higher risk was observed with respect to the regional figures [19]. Moreover, in the last years, the highest rates of mesothelioma mortality are attributable to people born between 1930 and 1949 who experienced the highest occupational exposure after the Second World War, prior to the 1992 asbestos ban.
- Updating the Italian national surveillance system of mesothelioma incidence cases: from 1993 to 2021, 37,003 mesothelioma cases have been collected by ReNaM. The modalities of exposure to asbestos were investigated for 29,020 (78.4%) cases, including 19,982 (68.8%) with occupational exposure. This percentage was not constant geographically and showed great variability.
- Estimating the impact of asbestos exposure on the development of ovarian cancer in Lombardy between 2000 and 2018: out of a total of 10,462 cases of ovarian cancer, 574.5 are attributable to asbestos exposure, with a higher percentage in municipalities highly impacted by asbestos [34].
- Ongoing update of the questionnaire used for the detection of mesothelioma cases by the COR‑ReNaM.
- Developing a model for psychological support to former asbestos‑exposed workers and their families: the model applied in the Casale Monferrato community, highly exposed in the past to asbestos, because of the presence of a large asbestos‑cement plant, highlighted the different steps of the intervention [35, 36].

### Participative initiatives and communication modality

The communication modality needs to be suitable for the proactive engagement of social stakeholders, ensuring their active role in participative initiatives. This is essential to allow the social stakeholders to constructively influence the project activities, going well beyond the simple passive role of being informed by researchers. The selected topics must be presented in a language that is suitable for the exchange of scientific information and data and that is tailored to the specificities of the event and the audience. The dialogue‑oriented approach of the SEPRA project and its objectives have been shared with the involved social stakeholders to foster their proactive participation. The key methodological aspect concerns the attempt to enable engaged stakeholders to influence the project activities, not simply being influenced by the project outcomes. After the presentation of the main topics and the preliminary results of the project by the researchers, social stakeholders shared their notions and suggestions, and the implications in public health were discussed during a participative workshop by researchers and stakeholders.

### Collecting inputs and enabling impacts

The participative initiatives need to be designed to facilitate the input collection from social stakeholders. Inputs need to be analysed to enable a constructive impact on the project and shared with the social stakeholders. This implies that researchers are fully aware of the importance of participative communication, which is a key element integrated into the project. Inputs collected during the project lifetime, together with the analysis of their impact on ongoing project activities, have to be evaluated at the end of the project to assess the overall impact of communication activities (that is, the fourth step of the adopted communication approach). With this aim and to provide indications for the future actions during the SEPRA project, the contributions and inputs provided by the social stakeholders have been collected by two researchers (DM, LF) separately, taking into account the experiences and skills representative of the selected categories. After the workshop, the collected information has been jointly analysed by the two researchers. On that basis, the main points and feedback from the participated interactions between researchers and social stakeholders have been discussed and agreed upon by all the authors.

## Results

The engagement of the selected social stakeholders was fruitful. Three social stakeholder categories participated in the workshop, for a total of 17 representatives. The participative modality of the workshop allowed the involvement of researchers and stakeholders in sharing data and information on the project’s activities and discussing the preliminary results of the project and its implications in public health and social initiatives.

At the beginning of the workshop, the dialogue‑oriented approach adopted in the project was presented to the participants, together with the workshop objectives. Then, the project researchers illustrated the selected research topics, using slides with accessible graphic representations and lay language, as well as providing updated information and tailored messages to the participants. A highly participated collective discussion characterized the second part of the workshop, which fostered multiple interactions among participants and researchers.

### Major points of concern and inputs from social stakeholders

The social stakeholders requested in‑depth information on mesothelioma mortality and incidence cases, as well as expressed concerns on the impact of the other ARDs. They provided inputs regarding the improvement of tools commonly used for prevention actions, assistance and support for former asbestos‑exposed workers, the population living in asbestos‑contaminated areas, and patients and their families with ARDs.

Social stakeholders were very concerned about asbestos risk and the health‑related impact of widespread non‑occupational exposures, particularly in populations who reside in asbestos‑contaminated sites less studied to date, in a perspective of an updating of the list of contaminated sites of national concern for remediation. Moreover, the need to recognize all ARD cases, in addition to mesothelioma, in all actions against asbestos impact, from epidemiological surveillance to health support and welfare, was highlighted.

Social stakeholders emphasized the need for a national plan addressing the psychological support to patients and their families affected by mesotheliomas, relying on the successful model and practical experience undertaken in the Casale Monferrato asbestos‑contaminated community. They pointed out the relevance to training the key health professionals and operators, including the general practitioners, to foster their awareness of asbestos risk and health impacts on the exposed population as well as to strengthen their involvement in sharing their experiences on ARDs with the affected patients.

From their practical experience in supporting workers and the affected population to obtain appropriate assistance, insurance, and welfare for asbestos‑exposed people, social stakeholders highlighted the necessity of major cooperation among the relevant institutions committed to the recognition of all ARDs. From their viewpoint, this highlighted the challenge for the national authority and its regional‑local offices to collaborate and work homogeneously.

Table 1 summarizes the discussed research topics and the social stakeholders’ inputs during the participative workshop, distinguishing between issues concerning the project’s activities and those related to the themes but outside the roles and responsibilities of involved institutions. The former will feed shared project’s recommendations that will be discussed with and delivered to institutional stakeholders (second column). The latter will result in project dissemination to policymakers (third column).

**Table 1:** Discussed research topics and social stakeholders’ inputs on project activities. The third column shows the requests made by social stakeholders on issues related to the discussed research topics, but outside the roles and responsibilities of involved Institutions. They will be disseminated by the SEPRA project to policymakers.

DISCUSSED RESEARCH TOPICS	SOCIAL STAKEHOLDERS’ INPUTS TO IMPLEMENT PROJECT ACTIVITIES	SOCIAL STAKEHOLDERS’ REQUESTS TO POLICYMAKERS
Sharing the dialogue‑oriented approach to foster proactive participation and the project’s implications in public health.	Strengthening their role in collaborating with researchers. Improvement of tools commonly used for asbestos prevention actions.	Revision/definition of the asbestos‑contaminated sites of national concern for remediation. Implement environmental remediation of asbestos‑contaminated areas, and removal of asbestos containing materials. Improvement of social insurance and welfare for all occupational and non‑occupational victims of asbestos, with the recognition of all ARD cases. Implementation of health surveillance plans at the national scale.
Updating the epidemiological surveillance of mortality from mesothelioma in Italy at the municipal level.	Strengthening the study of asbestos non‑occupational exposures at the municipal level. Estimates of mortality from ARDs, different from mesothelioma. Forecasting ARDs’ impact.	
Updating the Italian national surveillance system of mesothelioma incidence cases.	Improving the territorial homogeneity in the recognition of mesothelioma cases and asbestos exposure contexts. Estimates of ARD incidence cases different from mesothelioma.	
Updating the questionnaire used for the detection of mesothelioma cases by the COR‑ReNaM.	Recognizing all ARD cases, in addition to mesothelioma. Strengthening information exchanges between COR‑ReNaM technicians and social stakeholders for ARD case detection. Interactions on updating questionnaire.	
Estimating the impact of asbestos exposure on the development of ovarian cancer in Lombardy.	Estimating malignant cancer cases attributable to asbestos, in addition to mesothelioma, in areas highly impacted by asbestos of the national territory.	
Applying the model for psychological support to former asbestos‑exposed workers and their families developed for the Casale Monferrato asbestos‑contaminated community.	Developing a national plan addressing the psychological support to patients and their families affected by mesotheliomas. Training key health professionals and operators, including the general practitioners on asbestos health impact to share experience on ARDs with the affected patients.	
Network of Institutions committed to the recognition of ARDs.	Improving cooperation between the national authority and its regional‑local offices, committed to the recognition of all ARDs.	

## Discussion and Conclusions

The experience described in this paper allows us to emphasize the mutual benefits for researchers and social stakeholders resulting from effective engagement and qualified interactions. They rely on a dialogue‑based approach, bi‑ and multi‑directional communication, and mutual recognition of different roles and responsibilities. Although the evaluation of the overall results of the project communication activities will be carried out at the end of the project, we believe that the discussion on modality and results of involving social stakeholders at mid‑term of project activities, with implications for the prevention of ARDs at the national scale, is useful for other asbestos research initiatives, and for different contexts of exposure to hazardous substances.

The emerging need to strengthen collaborations and exchanges among all the involved actors in the chain, from researchers to institutional and social stakeholders, arose during all the interactions we have had so far in the project. The national, regional, and local health institutions committed to asbestos prevention, environmental remediation, health surveillance (screening, diagnosis, and treatment), including social and psychological support, social assurance, and welfare, represent a knot of this chain. General practitioners represent the first step in the relationship with patients and should be mainly committed to the issue of the ARDs, including the rights of the patients and their families.

Some concerns raised by social stakeholders during the interactions with researchers are not directly included among the responsibilities of the researchers and institutions participating in the SEPRA project, i.e., the definition of the contaminated sites of national concern for remediation, or the implementation of health surveillance plans. Nevertheless, these concerns should be highlighted by researchers in future communication initiatives of the project’s results (Table 1). With regard to health surveillance in Italy, regional health surveillance plans for former asbestos‑exposed workers have been established by law since 2008 (D.lgs. 81/2008) and are envisaged by the National Prevention Plan 2020–2025, but their implementation is still fragmented and not homogeneous across the country. They are included among the minimum healthcare and assistance actions (LEA) provided by the local Health Units. Social stakeholders pointed out their inhomogeneous implementation in the national territory and the need for their reinforcement. The urgency to implement health surveillance plans in all the regions of the country to tackle regional gaps and delays has been acknowledged by the SEPRA project.

The public health institutions participating in the SEPRA project, i.e. the National Institute for Health and the ReNaM, have committed to push local and national health services to work in this direction. Furthermore, it could be useful for patients, workers formerly exposed to asbestos and their associations to engage with the COR‑ReNaM to foster synergy and achieve a participatory surveillance (Table 1).

Another topic that emerged from the interactions with social stakeholders was the estimates of ARD cases different from mesothelioma. These activities are already planned in the project. The case study of the estimates of ovarian cancer deaths attributable to asbestos in the Lombardy Region was published in 2024 and presented during the workshop [34, 37]. The possible application of the applied model in other contexts, such as the implementation of an ad hoc model for lung cancer, will be addressed by the researchers in the next stages of the project, also considering the implications for assurance assistance and welfare actions highlighted during the discussions.

Furthermore, the issue of the recognition of ARD cases due to environmental exposure to asbestos also emerged during the interactions. The recent update of mesothelioma mortality epidemiological surveillance, performed by the National Institute for Health, focused on the subpopulation ≤50 years old as an indicator of environmental exposures in the child–adolescent period [12]. The revision of the questionnaire used by COR‑ReNaM, an ongoing activity in the SEPRA project, will consider this issue with particular attention, also with the involvement of the social stakeholders as they requested.

In Italy, one‑time financial assistance for all individuals affected by mesothelioma, beneficiaries of an ad‑hoc pension, is established by law (Law 244/2007). The establishment of a specific fund for the mesothelioma patients (and their families) who have suffered non‑occupational exposures, introduced since 2015 (Law 190/2014), is a clear expression of the potential connection between the epidemiological research and the public health policies in the Italian factual context [38]. Social stakeholders highlighted the urgency to enlarge the financial support to non‑occupational cases, as well as to the other ARDs, as required by law since 2021 (Law 178/2020). In this perspective, two actions should be undertaken. On the one hand, improving awareness of the affected citizens of their health rights concerning non‑occupational exposure to asbestos and including this issue in the questionnaire proposed to patients by ReNaM. On the other hand, major efforts to simplify the institutional procedures for the recognition of occupational and non‑occupational exposure to asbestos are needed. These actions are in line with the resolution performed by the European Parliament on the recommendation for protection of the workers from asbestos, drawing attention to both occupational and environmental asbestos exposure, including natural asbestos fibres, and all ARDs (P9_TA(2021)0427; available on: https://www.europarl.europa.eu/doceo/document/TA-9-2021-0427_IT.html).

The need to increase the awareness of the affected people and their general practitioners on their rights was another issue emphasized by social stakeholders. General practitioners need training to increase their knowledge on recognizing ARD symptoms, and to guide the patient towards early diagnosis, likewise in the occupational disease notification process [39]. Furthermore, the interactions between the epidemiological evidence and the medico‑legal issue still remain a great concern. From an insurance and medico‑legal point of view, it is essential to demonstrate the causal link between asbestos exposure and the occurrence of ARDs at the individual level and this is essential for the right to the recognition of financial support and welfare. On the other hand, the epidemiological findings provide evidence from a collective point of view and are in the probabilistic form. The use of the epidemiological results (which always include a measure of uncertainty) in a medico‑legal context remains problematic in practice. The forthcoming activities in the SEPRA project will address this critical issue, engaging the stakeholders in future discussions and interactions.

Social stakeholders also pointed out the need for forecasting the impact of ARD cases in the next years to plan welfare and assistance assurance programs. Specific investigations on this issue are planned in the SEPRA project and will be addressed in the next stages.

In addition to the needs and requirements expressed by social stakeholders, the researchers and operators of COR‑ReNaM asked for collaboration from the engaged trade unions and associations aimed at identifying people formerly exposed to asbestos and people affected by ARDs. Social stakeholders agreed to develop interactions dedicated to identifying suitable ways to start collaborations on this specific action. All participants in the workshop agreed on the need to work together, pushing all local and national institutions to implement ad hoc actions.

Specific actions at the national level are required to reduce the existing territorial differences in ARD prevention, including the environmental remediation of asbestos‑contaminated areas. To this end, the updating of the operative tools of the epidemiological surveillance system (such as ReNaM national guidelines and questionnaire), underway within the SEPRA project, could contribute to improving the territorial homogeneity in the recognition of cases and exposure for the prevention policies. Furthermore, the update of the geographical distribution of mortality and the incidence of mesothelioma risk and the estimates of ARD cases in the different regions, both actions underway in the project, contribute to highlighting the areas and the communities for which the asbestos impact is still underestimated and require ad hoc interventions. Therefore, recommendations regarding all these issues will be endorsed in the different stages of the SEPRA project. Participative public health recommendations, shared between researchers and social stakeholders, for actions needed to eradicate the health impact of asbestos in the country and to implement health and social assistance for asbestos victims and their families, will be included in the final report of the project. They will be discussed with institutional stakeholders committed to asbestos strengthening bi‑directional communication and further interactions.

Effective public participation and transparency in decision‑making on environmental and health issues are critical preconditions for the successful implementation of public health actions [2]. In our perspective, the engagement of social stakeholders in asbestos research operating at the national scale goes in that direction and allows for participative public health recommendations in more equitable decision‑making processes.

Creating environmental awareness through education and joint actions by society, governmental and non‑governmental organizations is essential to link evidence‑based information and to implement tailored actions to eradicate asbestos health impact, both in the countries that banned asbestos and in those where asbestos use is still permitted [10]. “Stakeholders and institutions can play an important role for improving the sensitization regarding the rights of compensation benefits, ensuring the equity and the effectiveness of insurance, welfare and public health systems” [40]. From an ethical perspective, meaningful stakeholders’ engagement can strengthen social capital, enhance inclusive decision‑making, and promote equity [41, 10].

The analysis of the diverse collective experiences of asbestos‑contaminated communities in Italy, from virtuous stories to poorly effective ones, corroborates the relevance of community engagement to enable the proactive role of social stakeholders in contributing to achieving concrete objectives for reducing asbestos health impact, from environmental remediation, and for implementing health and social assistance for the victims. This, in turn, allows communities to act on their individual and collective social and health rights.

In this perspective, researchers committed to asbestos public research at the national scale have to closely collaborate with social stakeholders through structured interactions, mutual recognition and trusted relationships, considering their diversified fields of experience, their critical thinking and inputs. Giving recognition of their role and expertise and providing them appropriate tools to deal with the relevant authorities and the asbestos‑affected people (access to updated information, bi‑and multidirectional communication and training, national and local institutional frameworks for periodic and effective interactions) are key actions for effectively advancing in inclusive processes and health equity.
